# Trade-Based Estimation of Bluefin Tuna Catches in the Eastern Atlantic and Mediterranean, 2005–2011

**DOI:** 10.1371/journal.pone.0069959

**Published:** 2013-07-26

**Authors:** Antonius Gagern, Jeroen van den Bergh, Ussif Rashid Sumaila

**Affiliations:** 1 Institute of Environmental Science and Technology (ICTA), Universitat Autònoma de Barcelona, Barcelona, Spain; 2 ICREA, Barcelona, Spain; 3 Institute for Environmental Science and Technology (ICTA), Universitat Autònoma de Barcelona, Barcelona, Spain; 4 Faculty of Economics and Business Administration and Institute for Environmental Studies, VU University Amsterdam, Amsterdam, The Netherlands; 5 Fisheries Economics Research Unit, Fisheries Centre, University of British Columbia, Vancouver, Canada; Aristotle University of Thessaloniki, Greece

## Abstract

The Eastern Atlantic and Mediterranean stock of Bluefin tuna Thunnus thynnus (BFTE) has long been considered overfished and at risk of collapse. Although ICCAT quotas for this stock have decreased considerably over the past years, uncertainty exists about the degree of catch beyond this quota. The extent of such catch is an important piece of information in stock assessment models as well as being an indicator of the effectiveness of fisheries management. We present a model using Bluefin tuna trade data to infer actual catches. Basing our calculations on 25 countries involved in BFTE trade, we estimate that between 2005 and 2011, allowable quotas were exceeded by 44 percent. This gap between catch and quotas has slightly increased over past years, leading to estimated excess catches of 57 percent for the period between 2008 and 2011. To improve assessments, preparation and design of BFTE management, we suggest that the estimated total removals reported in this paper be included in stock assessment models for BFTE. An implication of our findings is that ICCAT member states should take stronger measures to monitor and enforce compliance with quotas.

## Introduction

Over the past decade, the Eastern Atlantic and Mediterranean Bluefin tuna stock (Thunnus thynnus, hereafter BFTE) has been brought to near collapse [1]. Reasons for this overexploitation are of both biological and anthropogenic nature. On the one hand, scientific understanding of population dynamics and stock recruitment has been limited. For example, we are only now starting to appreciate the degree of mixing between Western Atlantic and Eastern Atlantic stocks, as well as the possibility of a genetically distinct subpopulation in the Mediterranean [2]. Population assessments are therefore characterized by considerable uncertainty, particularly about estimates of spawning stock biomass. In addition to this scientific uncertainty, management has been unable to control fishing mortality, allowing this stock to fall to biologically precarious levels. Especially in the years leading up to 2007, the International Commission for the Conservation of Atlantic Tunas (ICCAT) routinely set quotas above the scientifically recommended ones, which were associated with maximum sustainable yield and instituted only weak enforcement of those quotas [1], [3] and [4].

With increased international pressure to improve management, in 2007 ICCAT started to put into place a set of more promising management measures. Since 2007, allowable quotas have been cut substantially, from 36,000 tons in 2006 to less than 13,000 tons in 2011. In addition, surveillance has improved and the Bluefin catch documentation (BCD) scheme was put in place to track BFTE along the entire supply chain and mitigate illegal catches.

Although these measures are promising as a means to help the stock recover, one major obstacle to successfully managing this species is the possibility of illegal catch, here defined as landings over and above allowable quotas. When setting a yearly quota, ICCAT bases its decision on the stock’s probability of recovery. Currently, the harvest control rule requires that the probability of recovery by 2022 is at least 60 percent [5]. However, the probability of recovery fundamentally changes with the assumption on excess catches, which, in the main model, is currently assumed to be zero. This assumption has been challenged in various studies basing their analysis on different indicators on illegal catch:

Basing calculations on vessel capacity and economic viability of the fleet, illegal catches were estimated to be up to 107 percent above allowable quotas in 2007 [3], and up to 60 percent between 2008–2010 [6]. Although based on solid extrapolations of available data, these estimates are indicators rather than direct measurements of illegal fishing. Since most BFTE is internationally traded, another promising approach has been to estimate catches through import and export data. The “Mind the Gap” report [7] is the latest study in this vein: Based on this study, illegal catches appear to have exceeded allowable quotas by 31, 75 and 141 percent for the years 2008, 2009 and 2010, respectively. On the other hand, while ICCAT’s Standing Committee on Research and Statistics (SCRS) acknowledges the significant catches beyond quota before 2007, it is the “Committee's interpretation […] that a substantial decrease in the catch occurred in the Eastern Atlantic and Mediterranean Sea in 2008 and 2009” as a result of a more stringent TAC (total allowable catch) setting process since 2008 and that overfishing after 2007 has dropped to negligible ( [8], page 82).

In this report, we build on, revise and update [7] to estimate illegal catches of BFTE between 2005 and 2011. We modify the various steps of the methodology used in this earlier study, perform a sensitivity analysis, and present the findings in a form which is relevant to ICCAT’s pending decision making about the future management of Atlantic Bluefin tuna.

### Data Used

#### 2.1 Trade data

All countries involved in legal BFTE trade keep detailed records of imported and exported goods, both in terms of quantity and value. The competent body for data collection usually is the customs agency or the national statistics agency, which in most cases makes trade data publically available, although often against payment. Beyond national statistical services, some intergovernmental organizations collect, and make available, regional statistical data. For the purpose of the present paper, monthly trade data for BFTE (between January 2005 and March 2012) were accessed through three sources: Eurostat, the official platform of European trade statistics provides all EU27 import-and export data in value and volume [9]; the Japanese customs agency; and GTIS (Global Trade Information Service), a provider of official national trade statistics. Trade data from all reporting countries specified by Eurostat and the Japanese customs data were included in the analysis. GTIS data was limited to the top trading countries representing 97.5 percent of both imports and exports of BFT. While Eurostat always reports data as provided by national statistical agencies, GTIS in addition contains customs data for some of the most important producing countries including Spain and France. All raw trade data analyzed in this paper are publically available. Although we used the service of GTIS for a subset of trade data, GTIS obtain its data uniquely from official, publically available sources of each reporting country. Import and export data are categorized into internationally harmonized 6-digit codes (HS codes) by statistical agencies, referring to specific commodities (e.g. “030345, Bluefin tunas Thunnus thynnus, Frozen) that may or may not be further itemized into nationally applicable subcategories based on 2- to 4-digit statistical codes. These 2- to 4-digit codes sometimes vary among countries and therefore cannot be directly compared between countries. These include, for example, the exact “presentation” of a traded product and allow distinguishing between fillets, gilled and gutted fish or unmodified, whole fish (e.g. for the United States “0303450000, Bluefin Tunas (Thunnus Thynnus), Frozen, Except Fillets, Livers And Roes”). In important importing countries, statistical codes are also used to distinguish between BFTE and other, similar Bluefin species (e.g. for the United States “Thunnus Orientalis (Pacific Bluefin Tuna), Frozen, Except Fillets, Livers And Roes”). In order to minimize the error resulting from inconsistencies between country-specific statistical codes, our data collection was conducted as follows:

- All trade flows corresponding to HS codes including “Thunnus thynnus” were selected.
- Whenever it was unclear whether a given trade-flow exclusively referred to *Thunnus thynnus* we dropped this trade flow entry, thereby underestimating overall catches by a probably small but unknown amount.
- Finally, based on trade statistics, Mexico and Panama apparently contribute to a significant part of BFTE export. However, these exports are likely to refer mostly to Western Atlantic Bluefin tuna or Pacific Bluefin tuna. We therefore dropped flows from Mexico and Panama.

**Figure 1:**
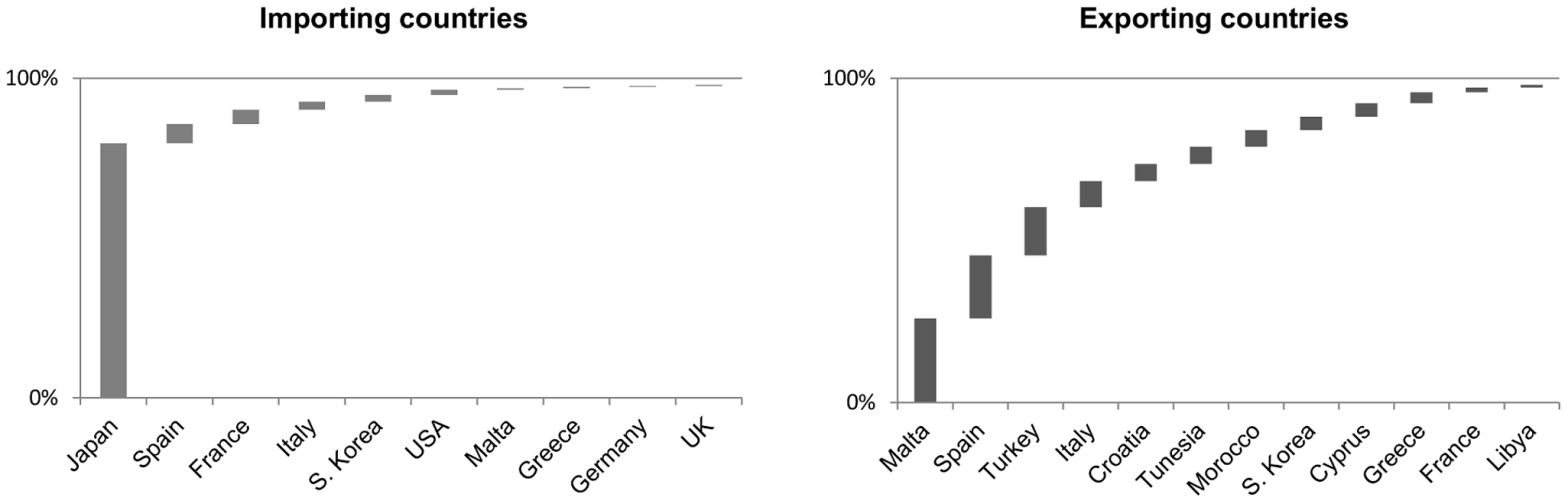
Main exporters and main importers of BFTE as reflected in trade data (traded product weight).

The raw data fed into the model (described below) finally covers 25 countries that exported and/or imported BFTE between the first quarter of 2005 and the second quarter of 2012. Just a few countries dominate this trade. Figure 1 shows the relative trade volumes of those countries that cumulatively account for 98% of import (10 countries) and export volume (12 countries).

Almost all countries report their trade data on a monthly basis (over 95 percent in volume). The rest is reported annually or quarterly. All trade flows were aggregated into quarterly imports and exports, in order to minimize the error in the crosscheck exercises (Section 3.2), while still allowing for the highest possible accuracy in adjusting time at trade to time at catch (Section 3.6).

### 2.2 Additional Data

The computation of fattening rates, corresponding to weight increase during a given fattening period (Section 3.5), required information on fishing gear, for which we consulted the ICCAT Task I database. The two main gears used in the BFTE fishery are Purse seine and Longline. While the latter is employed throughout the year, the former is limited to several weeks in late spring and early summer Purse seine catch (live BFTE) is transferred to fattening ranches (see Section 3.5). We therefore used the relative amount of catches harvested by purse seiners as the fraction of total catch that entered the fattening process each year. Formulas are given in Section 3.5. The ICCAT Task I database was further used as reference for recreational catches. Finally, we also use ICCAT conversion factors for round weight [10].

## Methods

**Figure 2:**
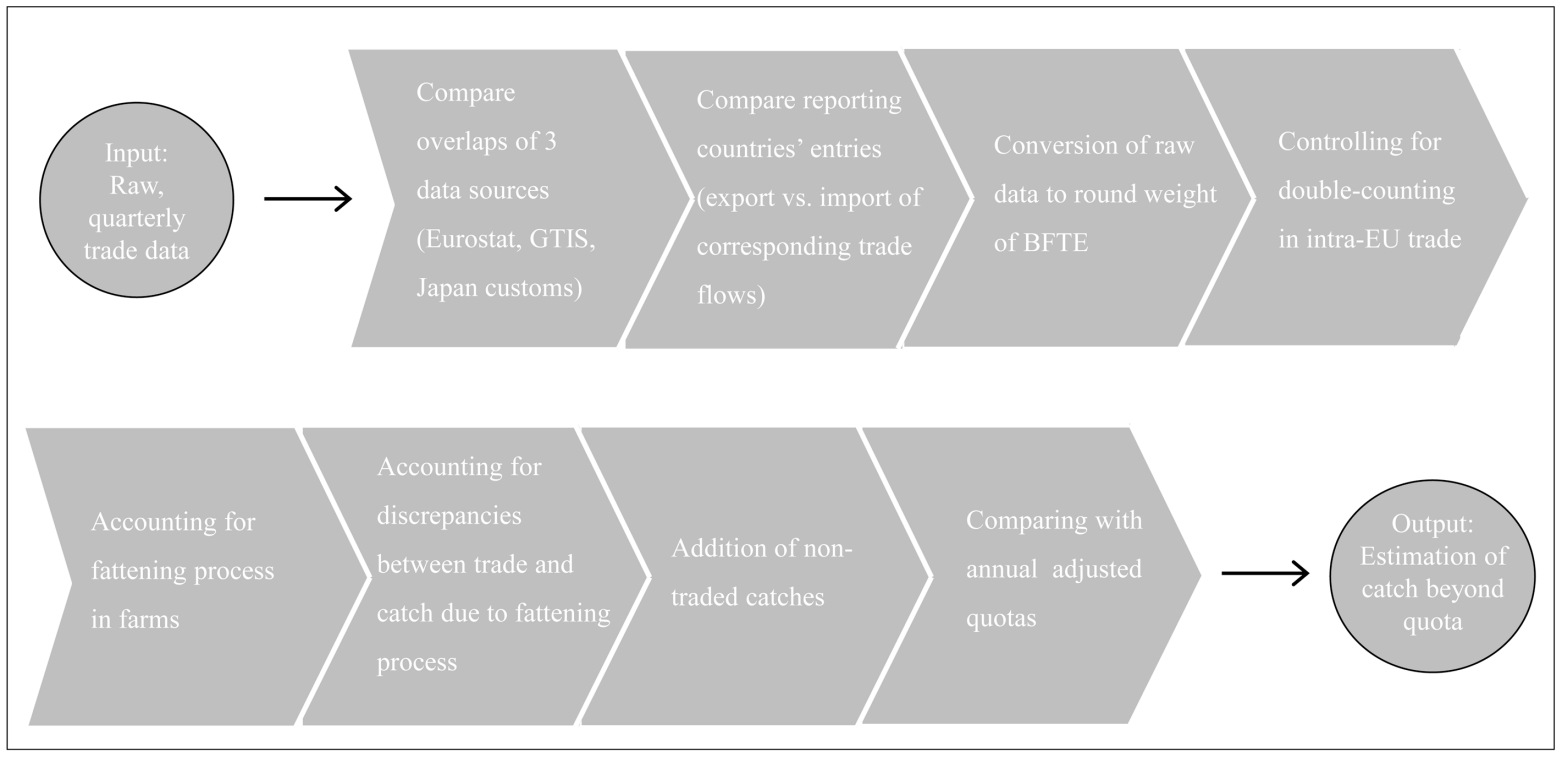
Graphical overview of the calculation approach.

Following a sequence of conversion calculations, the traded product weights as retrieved from the databases were transformed into live round weight at the time of catch and compared to annual allowable catch quotas. In the following subsections each step of the conversion is described in detail, from raw trade data to estimated weight at time of catch. A graphical overview of the calculation approach is provided in Figure 2.

### 3.1 Combining Data Sources

The three sources of data consulted cover distinct but overlapping sets of countries that report import from or export to partner countries. Together they represent the widest possible range of publically available data on BFTE trade. Following [7], we combined these data sets by comparing corresponding quarterly trade flows to avoid double counting. Whenever two overlapping data entries of distinct data sources conflicted, we picked the larger value in order to obtain the most complete data set and to detect inconsistencies between data sources. Anecdotal evidence suggests, for example, that the customs agencies of several European countries have underreported BFTE exports to the national statistics agencies and hence to Eurostat. As a result, one would expect Eurostat data to include lower values than GTIS data, which also include original customs data. In fact, while import data are very consistent across data sources, export data conflict in various occasions. However, conflicting overlaps yield minimal differences in total export weight (<4 percent).

### 3.2 Comparing Reported Imports with Corresponding Reported Exports

Traded freight logged by one country as export to a specific partner country should be consistent with reported associated imports by the partner country. For example, if Italy reports exporting 1 ton of Bluefin fillets to Japan in February 2009, Japan should report importing 1 ton of Bluefin tuna fillets from Italy in the same month. This consistency is often absent, for which there are five possible explanations:

- Most traded BFTE is transported by sea, from the Mediterranean to as far away as Japan, South Korea, or the United States. The time lag between logging a particular freight as an export upon departure and as import upon arrival might result in seemingly inconsistent data, if the exports are recorded in a different month or even quarter or year than the imports, i.e. if reference timing is used inconsistently. The EC user guide on statistics [11] notes that “… the reference period in theory is again the calendar month in which the goods are imported or exported. In practice, information is generally assigned to the month in which the customs authority accepts the declaration”. The definition of “reference timing” as the change of ownership is, however, impractical for “… those interested in the transport aspects of the data” because “it is believed that the definitions used generally coincide with the timing of ownership changes, although by no means always.”
- In principle, incentives to under-report trade flows exist for both importers and exporters. At the exporters’ end, under-reporting can mask the trade of catch that exceeds the national allowable quotas and would, if reported, lead to a cut in quotas for the subsequent year. At the importers’ end, customs agencies might collaborate illegally with cargo agencies and introduce part of the shipment into the black market, or seek to avoid tariffs.
- During shipment, freight can get lost, spoiled, or otherwise damaged (Pew 2011). If, as a result, freight is discarded in transit to avoid customs fees upon arrival for a good that cannot be sold, importing countries will report a lower weight than exporting countries.
- There are also measurement and logging inaccuracies. Sloppiness during measurement, logging, and extrapolation of product weight at the customs agencies can lead to differences in reported data.
- Different levels of detail in reporting of BFTE products might lead to underreporting (never over reporting) in some countries. One example is Bluefin fillets, which might be traded as “fish fillets” in one country (thereby escaping our filter) and as “BFTE fillets” in another country.

We established three scenarios (hereafter “input scenarios”) that estimate total trade flow. These scenarios are as follows:

- The maximum scenario: If two corresponding trade flows conflict, the larger value is adopted. This scenario allows us to eliminate intentional under-reporting to a large extent. However, this procedure introduces two biases, namely overestimation because of time lag of logging and overestimation through always favoring the positive error of measurement inaccuracies. If the identical freight is reported in different quartiles by exporters and importers, the “max” scenario might overestimate overall catches because the model picks the higher value (reported by exporters) in one quartile and the higher value (reported by importers) in the subsequent quartile.
- The average scenario: If two corresponding trade flows conflict, their nonzero-average is taken. This scenario mitigates the error of inaccurate measurement, as well as the error introduced through time lags, but it assumes that no intentional under-reporting exists.
- The import data scenario: Only import data are taken into consideration. This scenario assumes that there might be under-reporting at the exporters’ end, but that neither under-reporting at the importers’ end nor losses during the shipping process occur. As in the average scenario, the errors introduced due to time lag of logging are eliminated, and no under reporting is assumed. In addition, freight discarded before arrival is ignored.

### 3.3 Conversion to Round Weight

Between harvest and trade, BFTE is gutted, gilled, dressed, and/or filleted. These different types of fish products are called “presentations.” To make up for the weight loss during these steps, we have to convert product weight to round weight. This step requires two types of information, namely suitable conversion factors for each type of presentation and the relative composition of product presentations in the trade data. While conversion factors to round weight are readily available from ICCAT [10], composition of presentation in most national trade data is not detailed enough to directly apply conversion factors to raw trade data. Fortunately, the main importer of BFTE, Japan (around 80 percent of all imports), provides the highest level of detail for BFTE product type. We therefore calculate and apply a weighted average conversion factor to all traded BFTE based on the relative appearance of “presentations” (product types) in Japanese import data (Customs Japan). This is formalized in equation 1. In all formulas, variables are written in capital letters while parameters are written in lowercase. Exogenous variables are labeled with an over line. To simplify notation, time indices are omitted.
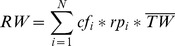
(1)where

***RW*** Round weight, which is the weight of the fish when taken out of the water, regardless of whether it has been ranched or not;
***cf_i_*** Specific conversion factor to round weight for “presentation” (ICCAT 2006). These factors are applied to traded product i indicates that these factors are presentation-specific (i.e. *i* denotes presentation);
***rp_i_*** Relative contribution of a given “presentation” in the Japanese import data;
***TW*** Traded product weight as specified in raw trade data.

Basing the conversion factor to round weight solely on Japanese import data might introduce an error if product types for Japanese markets significantly differ from those earmarked for other import markets. We therefore establish three values around the calculated weighted average as possible conversion factors.

### 3.4 Elimination of Double Counting within EU Trade and Estimation of EU Consumption

Two major constraints to the analysis apply to catch and trade within some of the main quota countries. First, it is not possible to capture locally caught and consumed BFTE through trade data as these catches are not reflected in trade data; second, it is not possible to distinguish exports from re-exports (for example, if Spain ships to France, which subsequently re-exports the product to Japan).

While this double counting problem caused by exports and re-exports applies mainly to France, Spain, and Italy (making up a “circular” trade representing around 13 percent of global imports, Figure 1), an inability to account for local consumption applies to all Eastern Atlantic and Mediterranean fishing countries (hereafter EU fishing countries) with BFTE quotas. We simultaneously controlled for both errors by replacing import entries of the EU block Spain, France and Italy with an estimate of BFTE consumed in all EU fishing countries. We do so by introducing a parameter (“EU consumption”) that represents a consumption ratio between the EU fishing countries and the three end markets of Japan, USA and South Korea, which together make up 85 percent of BFTE import between 2005 and 2011. The introduction of this parameter hence does two things: It eliminates all potential double counting due to re-export and it includes an estimate of consumption in Eastern Atlantic and Mediterranean fishing countries. Unfortunately, the scientific literature does not offer recent estimates on BFTE consumption that are independent of trade data. We therefore base our range of values on two types of information. First, we consulted online newspaper articles and NGO statements; second, we conducted five interviews with industry representatives, BFTE scientists and NGO representatives. Interviewees spoke to us under the premise not to be cited due to the politically tenuous nature of BFTE management in the past. These sources rather consistently point out that i) Consumption in Japan, the US and South Korea makes up about 80–90% and that the rising demand of high-grade sushi products in the EU has led to a higher presence of BFTE into local markets. In the model, we thus use 10, 15, and 20 percent (corresponding to 80–90% of consumption in the main end-markets) as possible values but select the most conservative value (10 percent) for a scenario that we highlight as the “preferred scenario” (see Section 4.6). The steps presented in (2) (defining end markets) and (3) (applying the “EU consumption” parameter) only change round weight entries for Spain, France and Italy, while other countries’ trade data entries remain unchanged.
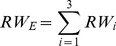
(2)




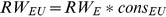
(3)where

***RW_E_*** Round weight of the main non-European end markets (Japan, South Korea and USA);
***RW_i_*** Individual round weight per non-EU end market country (Japan, South Korea and USA, denoted by subindex *i*);
***RW_EU_*** Round weight of the EU countries where circular trade and re-export can be expected (France, Italy and Spain);
***cons_EU_*** Consumption in France, Italy and Spain as a fraction of import going to the block Japan, South Korea and USA.

### 3.5 Conversion to Catch Weight

Net round weight does not always correspond to weight at catch. Some of the caught BFTE are transferred live into tuna ranches where fish are kept to reach the ideal fat content and meat color. During this process, BFTE also gain weight. To compare estimated catches with the allowable quotas, we must take such weight increases into consideration. This is addressed in two steps. First, trade flows are split into those with an origin in Croatia and those with another origin. Croatia is the main country entitled to catch BFTE at the minimum individual weight of 8 kilograms (As allowed for Adriatic catches), while the quotas of all other areas require a catch limit of 30 kilograms. This difference in catch weight fundamentally changes the assumptions related to fattening processes, given that wild juvenile fish have higher growth rates. Second, fattening rates are established to account for the weight increase during the ranching process.

#### Non-Croatian fattening

BFTE fattening in non-Croatian farms usually takes place between July and April. Although meat quality increases towards the winter, some fish are harvested throughout the rest of the fattening period in response to market dynamics and to avoid over-supply in the winter months. The best publically available set of data on non-Croatian fattening rates is presented by Galaz [12], spanning the period between 1995 and 2005, and including observations on more than 12,000 BFTE individuals. In this study, length frequency distributions (LFD, relative frequencies per size class) are presented, as well as cumulative size-specific fattening rates (weight increase per month and per size class between August and April) over the entire fattening period. LFD is crucial for the computation of fattening rates since different size classes have different growth patterns. Note, as opposed to natural conditions, young individuals in captivity can display high growth and fattening rates as long as they are the dominant size class in the pen; otherwise they seem to suffer from being underrepresented and grow even slower than mature, older fish [12]. We adapted the findings to calculate a weighted average fattening rate, which is then multiplied by the calculated net weight. Equation (4) yields the average monthly fattening rate, based on which equation (5) calculates the overall weighted average fattening rate. Equation (6) then applies this fattening rate to the purse seined fraction of non-Croatian net weight, to calculate catch weight before the ranching. Equation (7) is merely an auxiliary equation defining 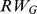, which is a variable appearing in (6).
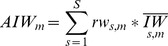
(4)




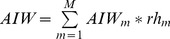
(5)

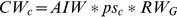
(6)




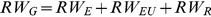
(7)where

***AIW_m_*** Average monthly (cumulative) increase of weight during the non-Croatian fattening process;
***rw_s,m_*** Relative weight of size class *s* in month *m* as compared to total weight in month *m;*
***IW_s,m_*** Increase in weight per size class *s* in month *m;*
***AIW*** Average increase in weight during the entire fattening process;
***rh_m_*** Relative harvest per month *m* during fattening process;
***CW_c_*** Estimated catch weight of non-Croatian fishing countries before any fattening process;
**ps_c_** The country-specific fraction of purse-seined catch as specified by the ICCAT Task I data base;
***RW_G_*** Global round weight excluding Croatia;
***RW_R_*** Round weight exported by countries not included in 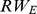 or 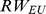 and excluding Croatia.

#### Croatian fattening

Croatian BFTE ranching is focused on smaller individuals, making the ranching time longer and fattening rates higher than in non-Croatian ranching. The studies [13]–[14] and [15] report a weight increase of over 500 percent for individuals that entered the pens between 6 and 8 kg (very small specimen) over a time period of almost 2 years, and weight increase of 220–320 percent for larger individuals. As none of these studies discloses LFD or even mean sizes of ranched BFT, it is difficult to make an assertion about Croatian weight increases during fattening processes. Furthermore, ([15], page 542) states that “since the rearing conditions are not fully controlled but depend on environmental changes, these indications should not be used for back-calculations [inferring from round weight to catch weight before fattening] to determine the initial quantity of fish stocked into cages.” Finally, some of the ranched fish in Croatia originates from other countries including Italy and France, where legal catch sizes start at individuals >30 kg and whose ranching yields similar weight increases as non-Croatian fattening rates. We therefore propose three estimates (2, 2.5 and 3) of Croatian fattening rates (CFR) so that the formula applied to Croatian exports becomes equation (8):
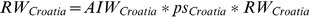
(8)where

***RW_croatia_*** Round weight exported by Croatia;

**AIW_Croatia_** Average cumulative increase of weight during the Croatian fattening process;

**ps_Croatia_** The Croatian fraction of purse-seined catch as specified by the ICCAT Task I data base;

**RW_Croatia_** Round weight exported by Croatia.

### 3.6 Weight at Time of Catch

Allowable quotas have greatly varied over the past years. When comparing trade data with quotas we therefore must correct for the time lag introduced by ranching. Bearing in mind that the main fishing season takes place between June and July and the fattening process stretches at least into April, we attributed all trade between January and June (quarter 1 and quarter 2) to catches from the previous year. Beyond that, we date all exports coming from Croatia back another 2 years, acknowledging the longer duration of the fattening process in that country.

In order not to underestimate Croatian catches in 2010 and 2011, an auxiliary set of export data was created for Croatia covering the years 2012 and 2013, as well as the first two quarters of the year 2014, based on average Croatian exports of the past 3 years. This might slightly overestimate the fraction of Croatian catches between 2009 and 2011 since quotas have been falling over past years. Similarly, we created a set of data for non-Croatian trade data for the second quarter of 2012, based on average values on the second quarter of 2009, 2010 and 2011. This again might lead to a slight overestimation of landings if catches have fallen as much as quotas have been falling in this time period.

### 3.7 Addition of Non-traded Catches

Part of the allowable quotas is earmarked for recreational fishing but cannot be traded and is thus not captured by the trade analysis [7]. This recreational fishing data were added without modifying weight. Landing figures are assumed to reflect round weight.

### 3.8 Sensitivity Analysis

Based on the different values of each variable that we considered in the model, a simple linear sensitivity analysis of all uncertain parameters was conducted. To do this, a total of 243 gaps (illegal catch as a percentage of allowable quotas, hereafter referred to as “gaps”; three input scenarios and four variables with three values each = 3^5^ = 243 gaps) were calculated. These gaps refer to cumulative estimated illegal catch as a percentage of cumulative allowable quotas over the period 2008–2011, the period for which SCRS believes there is no fishing beyond the allowable quota.

Table 1 summarizes the gaps that were calculated based on the three input scenarios (maximum, average, or import data) and the different values that we attributed to the variables (fattening rates, EU consumption, and conversion factors) of the model.

**Table 1:** Calculated gaps based on all possible combinations of variables used in the model; FR = fattening rate, Rwt = round weight.

Maximum scenario		10% EU consumption			15% EU consumption			20% EU consumption		
FR Med	FR Croatia	Conversion to Rwt 1.4	Conversion to Rwt 1.45	Conversion to Rwt 1.5	Conversion to Rwt 1.4	Conversion to Rwt 1.45	Conversion to Rwt 1.5	Conversion to Rwt 1.4	Conversion to Rwt 1.45	Conversion to Rwt 1.5
	2	57%	63%	69%	64%	70%	76%	72%	78%	84%
1.15	2.5	55%	60%	66%	62%	67%	73%	69%	75%	81%
	3	53%	58%	64%	60%	65%	71%	67%	73%	78%
	2	55%	60%	66%	62%	68%	73%	69%	75%	81%
1.2	2.5	52%	**57%**	63%	59%	65%	70%	66%	72%	78%
	3	50%	56%	61%	57%	63%	68%	64%	70%	75%
	2	50%	56%	61%	57%	63%	68%	64%	70%	75%
1.3	2.5	48%	53%	58%	54%	60%	65%	61%	66%	72%
	3	46%	51%	56%	52%	58%	63%	59%	64%	70%
**Average scenario**		**10%**			**15%**			**20%**		
**FR Med**	**FR Croatia**	**1.4**	**1.45**	**1.5**	**1.4**	**1.45**	**1.5**	**1.4**	**1.45**	**1.5**
	2	10%	14%	18%	15%	19%	23%	20%	24%	29%
1.15	2.5	8%	12%	16%	13%	17%	21%	18%	22%	27%
	3	7%	11%	15%	12%	16%	20%	17%	21%	25%
	2	8%	12%	16%	13%	17%	21%	18%	22%	26%
1.2	2.5	7%	10%	14%	11%	15%	19%	16%	20%	24%
	3	5%	9%	13%	10%	14%	18%	15%	19%	23%
	2	5%	9%	12%	10%	14%	18%	15%	19%	23%
1.3	2.5	3%	7%	11%	8%	12%	15%	13%	16%	20%
	3	2%	6%	9%	7%	10%	14%	11%	15%	19%
**Imports scenario**		**10%**			**15%**			**20%**		
**FR Med**	**FR Croatia**	**1.4**	**1.45**	**1.5**	**1.4**	**1.45**	**1.5**	**1.4**	**1.45**	**1.5**
	2	−5%	−1%	2%	−1%	3%	6%	4%	7%	11%
1.15	2.5	−6%	−3%	0%	−2%	2%	5%	2%	6%	9%
	3	−7%	−4%	0%	−3%	1%	4%	1%	5%	8%
	2	−6%	−3%	0%	−2%	1%	5%	2%	6%	9%
1.2	2.5	−8%	−4%	−1%	−4%	0%	3%	1%	4%	8%
	3	−9%	−5%	−2%	−4%	−1%	2%	0%	3%	7%
	2	−9%	−6%	−3%	−5%	−2%	2%	−1%	3%	6%
1.3	2.5	−10%	−7%	−4%	−6%	−3%	0%	−2%	1%	5%
	3	−11%	−8%	−5%	−7%	−4%	−1%	−3%	0%	4%

Note that this table consists of three identically arranged sub-tables differing only with respect to input scenario: Maximum, Average and Imports. Percentages indicate the extent by which allowable quota have been over- or under fished between 2008 and 2011.The bold number (57%) is the gap calculated based on the *preferred* scenario, that is, values we regard as being most probable.

## Results

### 4.1 Comparing Reported Imports with Corresponding Reported Exports

**Figure 3:**
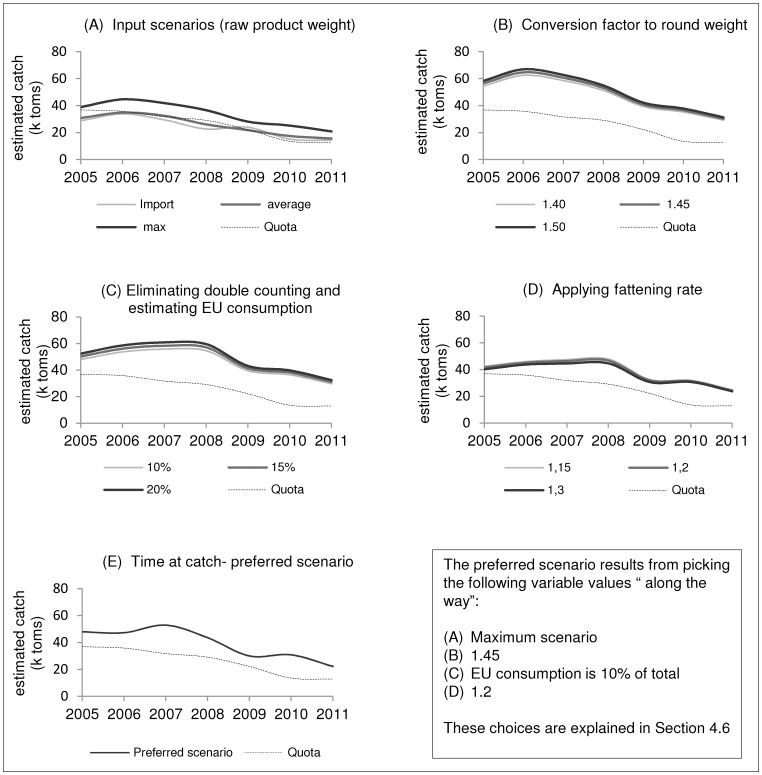
The development of estimated catch over the various stages of the methodology. B is based on maximum scenario, C through E are based on middle value of previous step.

The choice of the input method is a decisive step in this methodology. Using the Maximum scenario shows markedly higher overall catch estimates than the *import* or *average* scenarios (Figure 3A). This indicates the magnitude of inconsistencies in the reported trade data. It should be noted that Figure 3C through 3E always adopt the middle value and ignore the upper and lower bound of the previous step. This is with the exception of Figure 3D which adopts the lower bound scenario of Figure 3C (10% EU consumption). Together, these choices lead to our “preferred scenario” (Figure 3E).

### 4.2 Conversion to Round Weight

Table 2 summarizes the commodity-specific round weight conversion factors as used by ICCAT, as well as the composition of commodity types to the highest possible detail, as presented by the Japanese customs data. While the conversion factor of 1.67 for fillets (representing 65 percent of product weight entering Japan) is uncontroversial, it is less clear what round weight conversion factor to apply to the remaining 35 percent of product weight, which is solely designated as fresh or frozen Bluefin tuna (the descriptions in Japanese customs data offer slightly more detail, but they do not allow for more precise interpretation of the products’ presentation). Table 2 therefore also presents a set of weighted average conversion factors that are based on different assumptions pertaining to the presentation of the 35 percent of product weight that is unspecified. If we assume that all tuna of unspecified presentations have been neither gilled nor gutted, nor otherwise modified, we get to an overall conversion factor of 1.43. If we assume that all such unspecified products are in fact fully “dressed” (gilled, gutted, partly beheaded and some of the fins missing), an overall conversion factor of 1.52 is calculated. Basing our conversion factor solely on Japanese import data we hence calculate conversion factors ranging from 1.43 to 1.52., whereby the lower bound is improbable given the unlikelihood of BFTE being exported without modification. As the 20 percent of remaining trade data might have slightly different presentation patterns than is favored in the Japanese market we have used three values (1.4, 1.45, and 1.5) as conversion factors in the model. Figure 3B illustrates the change in estimated catch as a function of these three values. This figure is based on calculations for which the maximum input scenario is adopted.

**Table 2:** Weighted average conversion factors (to round weight) calculated based on different assumptions on product presentation.

Commodity type	Japanese import weight in percentage (2005–2011)
Fillet, fresh or frozen	0.0001%
Fresh Fillet	0.0087%
Frozen Fillet	64.7%
Fresh unspecified	17.0%
Frozen unspecified	18.2%
**Weight type**	**ICCAT conversion factor**
Dressed weight (DWT)	1.25
Gilled and Gutted weight (GWT)	1.16
Fillet weight (FIL)	1.67
**Hypothetical presentation of "fresh" and "frozen" BFT**	**Weighted average calculated**
All whole (conversion factor 1)	1.43
All GWT	1.49
All DWT	1.52
One half GWT, one half DWT	1.51
One third GWT, one third DWT, one third unmodified	1.48

### 4.3 Elimination of Double Counting within EU Trade and Estimation of EU Consumption

**Figure 4:**
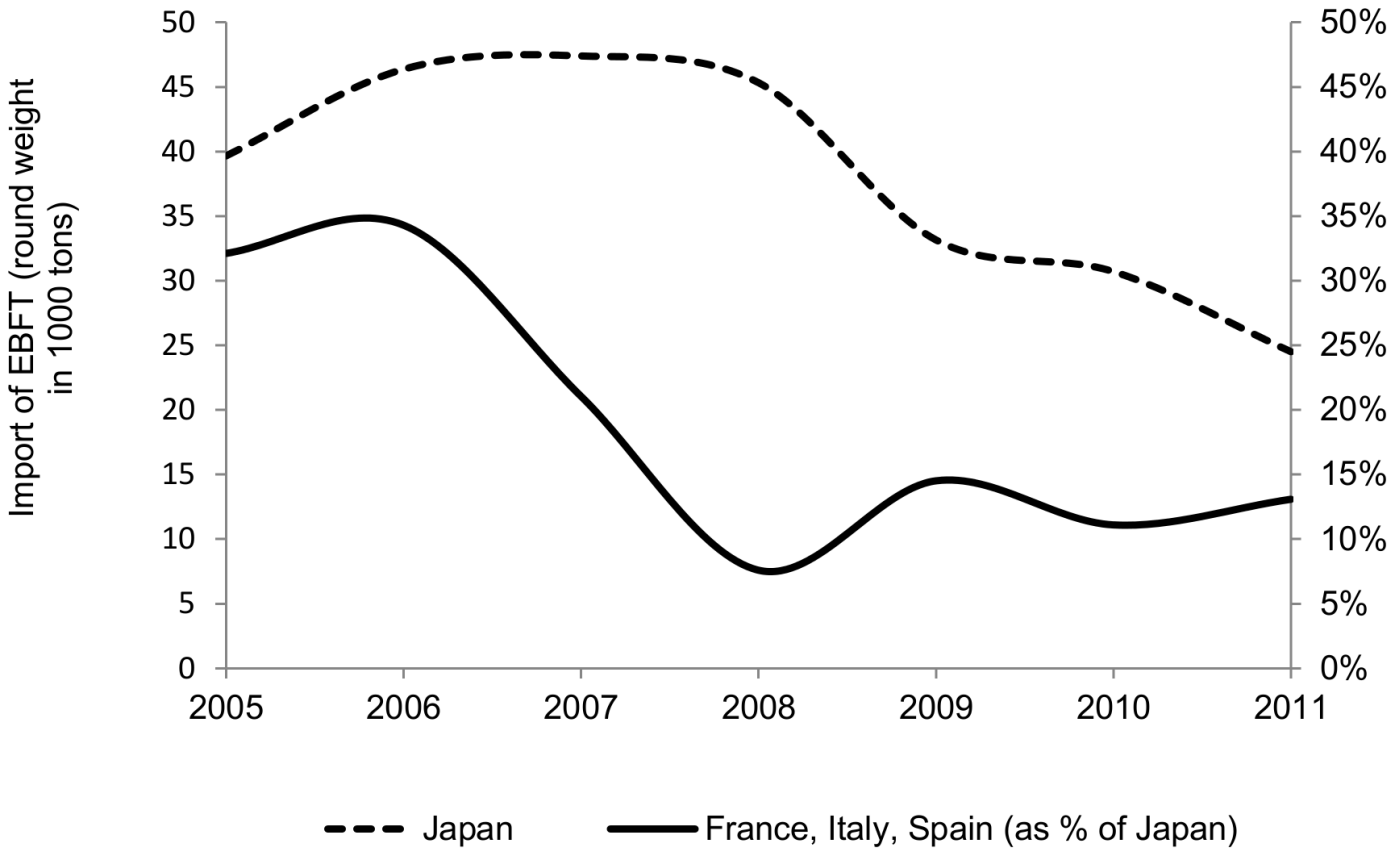
Import by main EU importers as a percentage of Japanese imports.

Based on the assumptions made on EU consumption, compared to *round weight* the calculated catch values are slightly lower until 2007 and slightly higher thereafter (Figure 3C). The reason for this is that French, Spanish and Italian imports of BFTE greatly decreased over the past years (Figure 4). Recalling our model specification on assumed EU consumption, this means that estimated round weight is corrected downwards as long as imports by France, Spain and Italy are higher than 10, 15 or 20 percent of global imports respectively, and upwards if the opposite is true. Interestingly, compared to *round weight*, the overall picture does not change much, suggesting that the positive bias of double counting is of similar magnitude as the negative bias induced by missing data on internal EU consumption.

### 4.4 Conversion to Catch Weight

**Figure 5:**
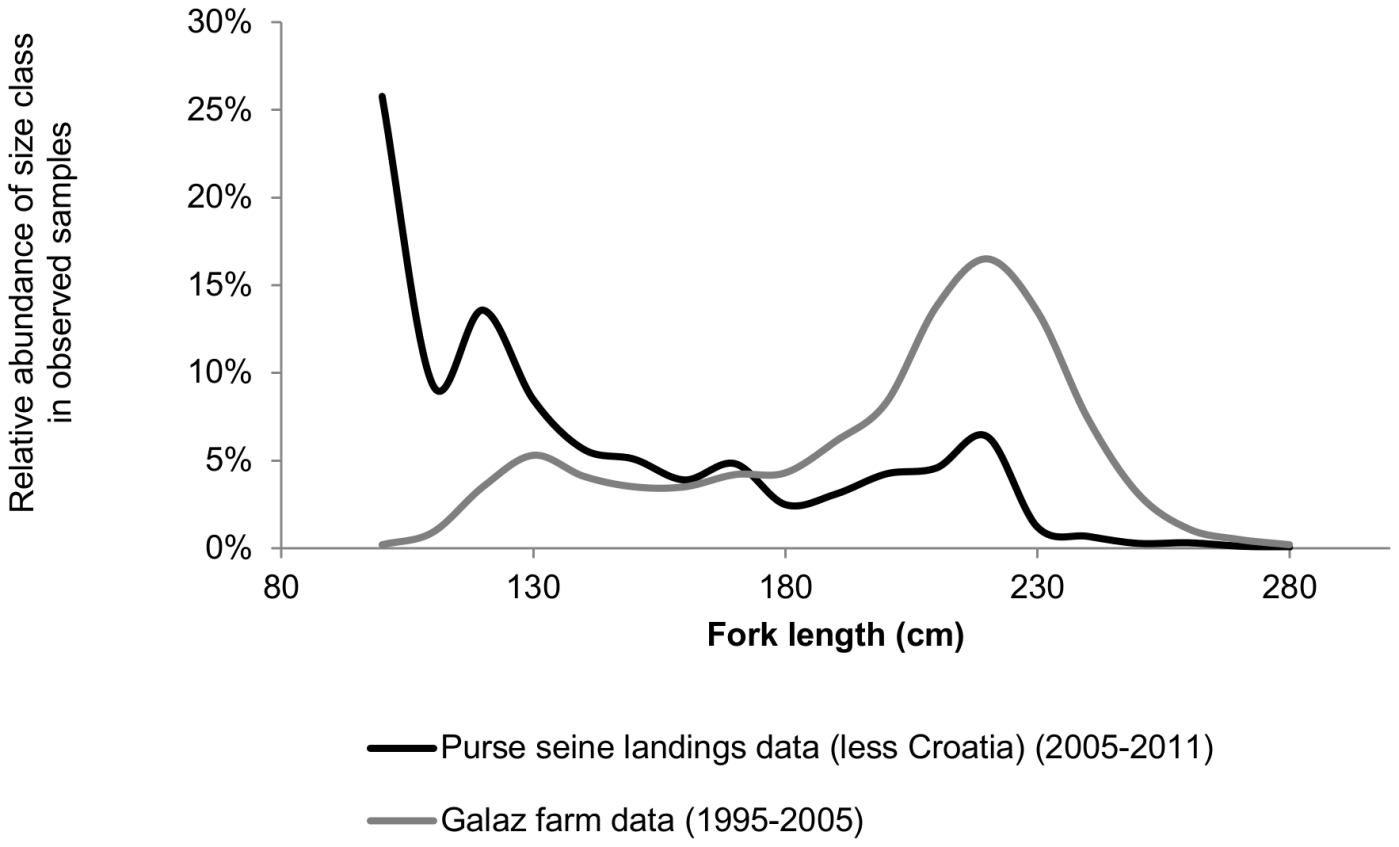
Length-frequency distributions based on different sources.

**Figure 6:**
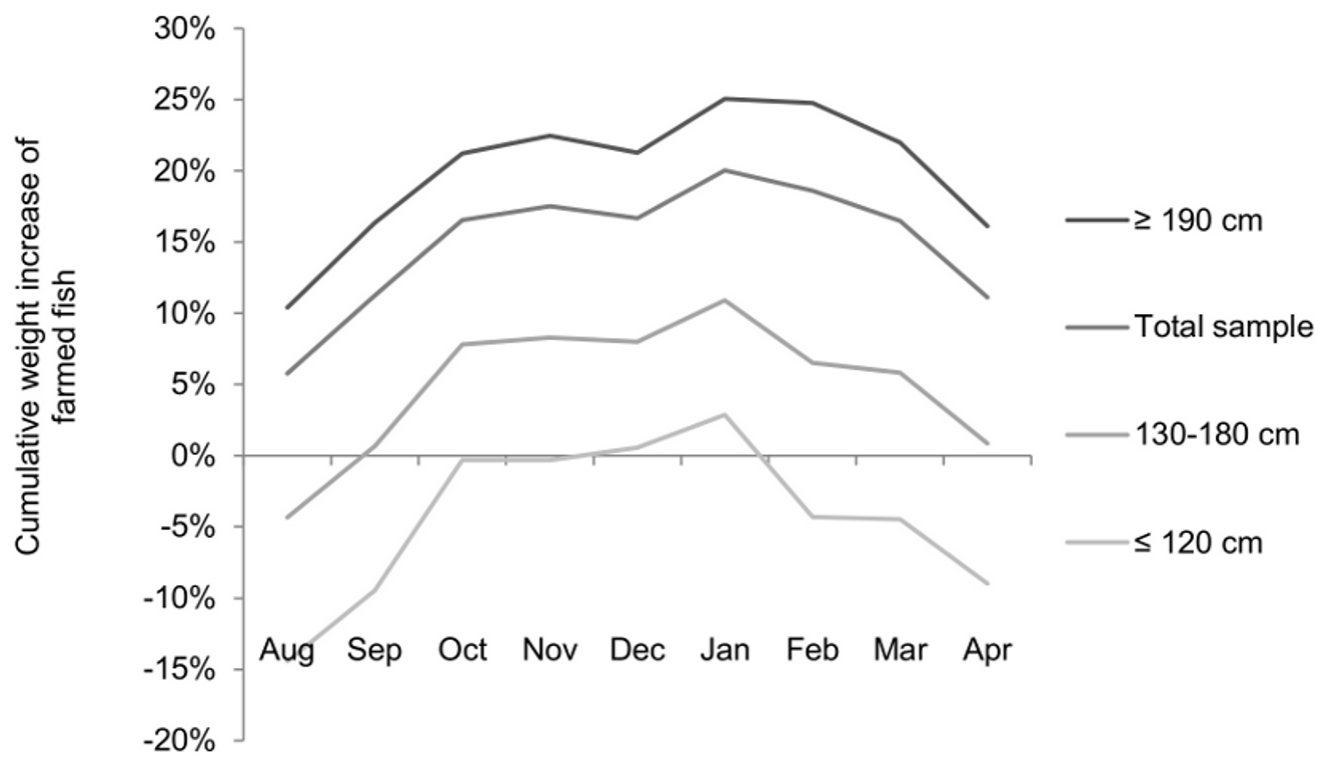
Size-specific cumulative weight increase during the period of non-Croatian fattening.

Combining the length frequency distribution (Figure 5) and size-specific cumulative rates of weight increase over the period of fattening (Figure 6), both based on [12], we calculated a weighted average fattening rate for non-Croatian BFTE farming of 1.16. Next to the LFD presented in [12], Figure 5 includes the LFD based on the purse seine catches (2004–2011) presented in the ‘ICCAT Task II size’ data base. As these LFD are fundamentally different from those presented in [12] we chose to include three values as possible non-Croatian fattening rates, namely 1.15, 1.2 and 1.3. However, contrary to our expectations, the choice of fattening rates has only a small effect on the estimated overall catch (Figure 3D).

### 4.5 Catch Weight at Time of Catch and Addition of Non-traded Catches

The reassignment of trade dates to catch dates pronounces the differences between catch seasons. The decline of estimated catch in 2009, followed by its sharp rise in 2010 (despite sinking quotas) suggests another dynamic being captured here, namely short term business decisions by ranchers (Figure 3E). Although we do not have specific data supporting this conclusion, it is reasonable to assume that the observed behavior is a consequence of rapidly sinking tuna prices in 2009, which caused tuna ranchers to keep their tuna in pens, waiting for the prices to stabilize again before selling (personal communication with an industry representative who prefers not to be cited here).

The addition of non-traded recreational catch increases the overall estimated catch by around 1 percent for the period of 2005–2011.

### 4.6 Defining a Preferred Scenario

The wide range of results of the sensitivity analysis (Table 1) does not represent equally probable outcomes. It reflects the model’s reaction to different values of the model parameters. Within the obtained range we would like to define a “preferred scenario” that we believe is the most likely. This scenario is based on the following assumptions and associated motivations:

- Given the high incentives to under-report trade data, as well as other dynamics favoring under-reporting, it seems legitimate to pick the maximum import scenario (Section 4.1).
- Basing the conversion factor for round weight on calculated weighted average values, a factor of 1.45 appears to be the most appropriate conversion factor while still permitting for some degree of conservatism (Section 4.2).
- Given the dearth of information on consumption and double counting, the value of 10 percent EU-consumption of BFTE was used as a conservative estimate (Section 4.3).
- The weighted average fattening rate calculated based on data from [12] suggests a rate of weight increase of 1.16 for non-Croatian ranches. Nonetheless, we favor the more conservative rate of 1.2 for two reasons. First, data used in that study cover the period from 1995–2005 and we can assume that fattening processes have improved since then. Second, although in [12] it is shown that small individuals increase in weight at a lower rate than large individuals, the difference in LFDs between Galaz [12] and the ICCAT data might suggest higher rates of weight increase given that ICCAT data show a predominance of small fish, a decisive factor for fish BFTE growth in fattening ranches [12]. Thus, factor 1.2 is used as a more realistic value. Given the shortage in publically available data on Croatian ranching, we prefer to choose the most conservative factor of 3 (Section 3.5 and 4.4).

**Figure 7:**
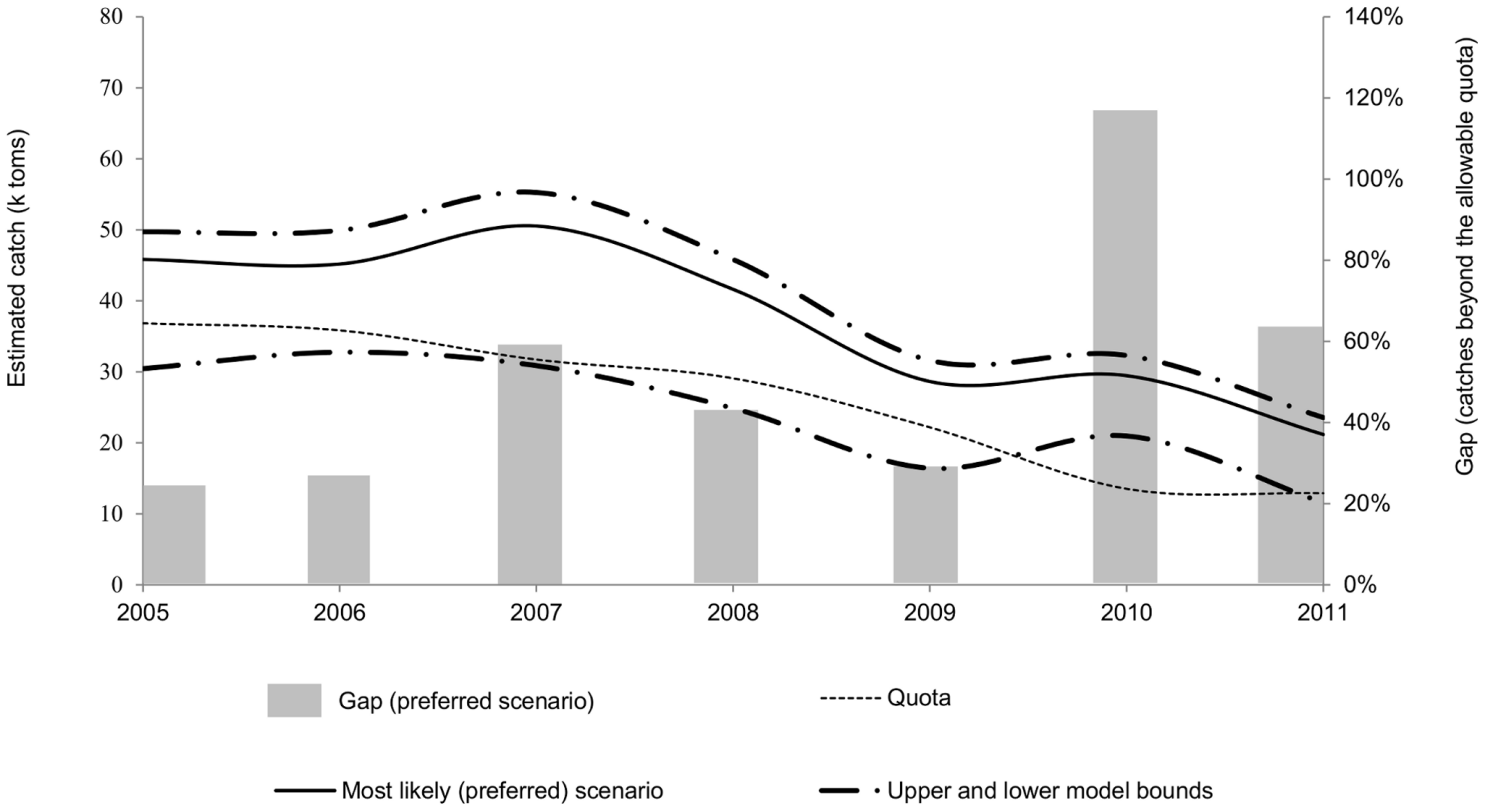
Estimated catches and corresponding gap (catches beyond quota).

Applying these assumptions, the model calculations suggest that between 2008 and 2011, total BFTE catches exceeded allowable quotas by 57 percent. The exceedance calculated for the years 2005–2007 is somewhat lower, namely 44 percent, because despite falling catches over past years, fishing quotas have fallen more rapidly than our estimates of catches. Figure 7 shows the upper and lower model bounds, highlights our “preferred scenario” and indicates the catch beyond quota (in percent) that is calculated based on this scenario.

### 4.7 Sensitivity Analysis

Table 1 shows that the highest sensitivity of the model is due to the choice of input scenarios (maximum, average, or import data). All other variables only lead to minor changes in estimated gaps.

## Discussion

Our study highlights significant levels of excess catch in the Eastern Atlantic and Mediterranean Bluefin tuna fishery. Providing a wide range of values for variables around which uncertainties exist, our findings show that one would have to take a range of highly questionable assumptions for granted to assume that no fishing beyond allowable catches has occurred between 2008 and 2011. These assumptions include that (i) no under reporting exists at the importers’ end, (ii) overall conversion factors from product weight to round weight are as low as 1.4, (iii) EU consumption of BFTE is merely 10 percent of overall consumption, and (iv) the highest fattening rates presented for both the Mediterranean farms and for Croatia are true.

Using the, in our view, most realistic values around each variable, cumulative illegal catch has exceeded allowable catch by 44 percent since 2005. As allowable quotas decreased over past years, and illegal catch did not decrease at the same pace, this figure rises to 57 percent of excess fishing for the period 2008–2011.

### 5.1 Possible Sources of Error

#### Data-related errors

We identified five potential sources of data-related errors, three of which would imply that we underestimate our final catch value and two of which would imply overestimating this value. First, the complete exclusion of non-quota countries can lead to some underestimation. [16], for example, suggests that between 2000 and 2010, 18,704 tons of Bluefin tuna (life weight equivalent) were traded via Panama without being reported to ICCAT. Second, our analysis does not capture catches that have been traded in black markets. This includes, but is not limited to, mislabeling, which can potentially take the form of downgrading (labeling BFTE as less costly fish to avoid citations of excess catch) and upgrading (labeling other tuna as BFTE to yield higher prices at end markets). Given the strict rules at customs agencies, the high price of BFTE, and the ‘connoisseur’-nature of end markets, upgrading can be expected to be minimal. Downgrading, on the other hand, is a common problem that has often been reported. The latest example includes the uncovering of 40 tons of BFTE labeled as yellowfin tuna and shipped from Italy to Spain in May 2012, representing 4 percent of Italian quotas for 2012 [17]. Third, the exclusion of trade entries containing other species than BFTE might lead to some underestimation. To the extent that data-related errors are concerned we are therefore confident that estimated excess catches presented in this study (the preferred scenario) are conservative. Fourth, Japanese Import data only poorly distinguish between Atlantic and Pacific Bluefin tuna. However, the countries considered as exporters do not, or only to a very small extent fish and trade Pacific tuna (See Figure 1 for reference). Fifth, before 2007, Inter-EU trade of life BFTE was poorly coded, potentially being partly included in the processed BFTE data. This error is not relevant for our main results, as these apply to the years 2008–2011.

#### Methodological errors

Such errors include the crosschecking both between sources and between reporting countries, the creation of auxiliary data sets to make up for recent years’ catch that has not yet been traded and, to a lesser extent, variable assumptions of our preferred scenario.

In our preferred scenario we always pick the larger of two values when conflicting entries arise. Although we believe that this is necessary to deal with under reporting, it unavoidably leads to overestimates. These have two origins. First, whenever a random deviation occurs in two corresponding entries, the positive deviation is favored and the negative error is dropped. Second, if there is a time lag between reporting export and reporting import, an error might be introduced if data entry is not identical to the date at which the product changes ownership. Since both errors are decreased at a higher degree of temporal aggregation of trade data, we used quarterly aggregation of data instead of monthly data.

The creation of auxiliary data sets for 2012, 2013 and 2014 is likely to overestimate total catches. This overestimation however is less severe than the one resulting from our crosschecking methodology. First, this overestimate only applies to Croatian exports, and within these exports only to the purse seined fraction of catches. Second, although quotas have decreased between 2009 and 2010, they stayed constant thereafter. Taking averages over the three-year period 2009–2011 thus leads to very low levels of overestimation.

Variable definition is a justified source of concern regarding the selection of our preferred scenario. However, as opposed to other errors herein presented, it is difficult to judge whether they tend to overestimate or underestimate the final results. On one hand, wherever data were poor we chose a more conservative variable value. On the other hand, extrapolations from Japanese import data could be misleading. This mainly pertains to the calculation of the conversion factor to round weight, which is a sensitive variable.

### 5.2 Comparison with Previous Studies

Similar to previous studies, this analysis confirms that illegal catch has been responsible for large parts of overall BFTE catches in past years. Although taking an alternative and significantly altered approach to calculate catches from trade data and despite fully independent data collection between the studies, our analysis largely supports the overall outcome of [6] and [7]: Illegal catch significantly and persistently surpasses current allowable quotas and this gap has been slightly increasing over past years in relative terms. This study adds three important dimensions to existing, published tuna trade analyses. First, we provide a mathematical model which converts raw trade data into catch estimates and presents each computational step in detail, thereby making the analysis transparent and reproducible. Second, we use monthly data aggregated into quarterly data instead of using annual data. This allows us to more accurately assign trade data to catch data and still avoid overestimations through time lags induced by shipment to distant destinations. Third, our model contains a detailed sensitivity analysis: We present estimates on illegal catch as a function of those variables in the model, around which some uncertainty exists; we then justify the use of a specific set of values for each variable both quantitatively and qualitatively, and define the in our view most realistic outcome for yearly excess catch.

### 5.3 Policy Implications and Recommendations

#### ICCAT quota

Currently, ICCAT uses size-structured population models to calculate the probability that, at a given catch, the stock recovers to MSY levels by the year 2022 [3]. Quotas are set at the highest level of catch that would still allow a 60 percent (or higher) probability of recovery. Using reported landings to estimate the levels of catch neglects illegal catch which, when included in the stock assessment models, is likely to result in incorrect quota levels. Although managers are provided with model outputs that include potential illegal catch, the main calculations are based on the assumption of zero illegal fishing. Including excess fishing in the model considerably decreases the probability of recovery at current quotas. We therefore urge ICCAT to include the estimates of 57 percent illegal fishing beyond actual allowable quotas when making decisions about future quotas.

#### Management at sea and in farms

Management of the Eastern Atlantic and Mediterranean Bluefin tuna keeps failing its objectives. Although quotas have been decreased, catch has not fallen anywhere close to desired values. As pointed out by previous research, insufficient enforcement of existing measures might have several reasons, most importantly weakly implemented BCD schemes [18], insufficient observer programs and low levels of cooperation among BFTE fishing countries [4]. To effectively tackle the problem of BFTE overfishing, these management tools must hence be strengthened and member states’ cooperation and accountability must be increased. However, as an important tool for successful management, a better understanding on the main source of incompliance must be fostered. Our analysis highlights that, smoothing the fluctuations of estimated catch between 2008 and 2011, excess fishing tends to adapt to allowable quotas. This might suggest that excess fishing is closely linked to unreported landings by vessels with quotas, and to a lesser extent with entirely illegal vessels. If this was the case, an increase of observer programs on vessels would have a significant effect on the mitigation of illegal catches. Although we cannot conclude this assertion based on available data, this represents one important question around illegal fishing and should receive more attention in future research.

Another weakness in the chain of management seems to be that some farms accept live BFTE from vessels not entitled to quotas (or only to a lesser extent than they supply). As farm operators are ultimately trading the BFTE, a swift improvement of the electronic version of the BCD scheme implemented should continue to be a high priority for ICCAT to allow effective and real-time tracking of all BFTE catches and to hamper black markets.
